# Micropropagation of *Pinus taeda* L. via axillary buds

**DOI:** 10.1186/1753-6561-5-S7-P144

**Published:** 2011-09-13

**Authors:** Leandro Francisdo de Oliveira, Luciana Lopes Fortes Ribas, Marguerite Quoirin, Henrique Soares Koehler, Antonio Rioyei Higa

**Affiliations:** 1 Programa de Pós-Graduação em Botânica, Departamento de Botânica, Universidade Federal do Paraná, Curitiba, Paraná, 81531-980, Brazil; 2 Departamento de Botânica, Universidade Federal do Paraná, Curitiba, Paraná, 81531-980, Brazil; 3 Departamento de Fitotecnia e Fitossanitarismo, Universidade Federal do Paraná, Curitiba, Paraná, 80210-170, Brazil; 4 Departamento de Ciências Florestais, Universidade Federal do Paraná, Curitiba, 80035-050, Brazil

## Introduction

*Pinus taeda* stands for productivity and quality of its timber [1]. Researches using biotechnology are of great importance and have been applied to the improvement of its timber and plantation [2]. The main method of *Pinus* propagation is by seeds, once the minicuttings depends on the season of the year or depends of juvenile material [3-5]. Thus, researches on micropropagation of Pinus taeda are currently a priority in Brazil [6]. Micropropagation is the best method for mass production of superior genotypes and represents a strategy for tree improvement and capture of genetic gains [7]. Studies on *Pinus taeda* micropropagation by axillary bud proliferation are quite few. The purpose of this study was to develop a protocol for micropropagation of this species from juvenile material.

## Materials and methods

For *in vitro* establishment two to four month old seedlings were used. Apical shoots and nodal segments of 3 cm length were inoculated in MS [8], DCR [9], WV3 [10] or WV5 [11] medium. For axillary shoots induction, the explants were inoculated in WV3, WV5 or DCR medium, with BAP (0, 0.12, 0.25 and 0.50 μM). For the induction of roots, we tested the effect of double-layer medium, with semi-solid phase consisting of agar and water or GDm/2 [12] medium and the liquid phase containing water or GDm/2 medium. Both phases were supplemented with 2.69 μM NAA and 0.44 μM BAP for 9 days, followed by transfer to growth regulator-free GDm/2 medium. The rooted plants were planted in Plantmax^®^ Forestry substrate and maintained in a greenhouse.

## Results and Discussion

**Figure 1:**
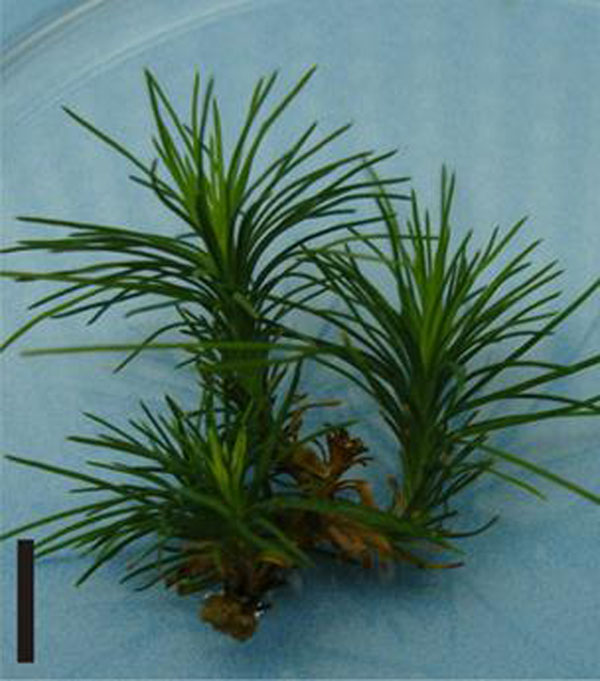
Nodal segments of *Pinus taeda* inoculated in WV5 medium, after nine weeks in the *in vitro* establishment. Bar: 1 cm.

Nodal segments showed better responses during *in vitro* establishment, with up to 100% of explants forming axillary shoots and an average of 4.3 to 5.8 shoots per explant. The WV5 media proved better and presented the highest survival rate (86.0%) and highest elongation percentage (85.2%) (Figure 1).

**Figure 2:**
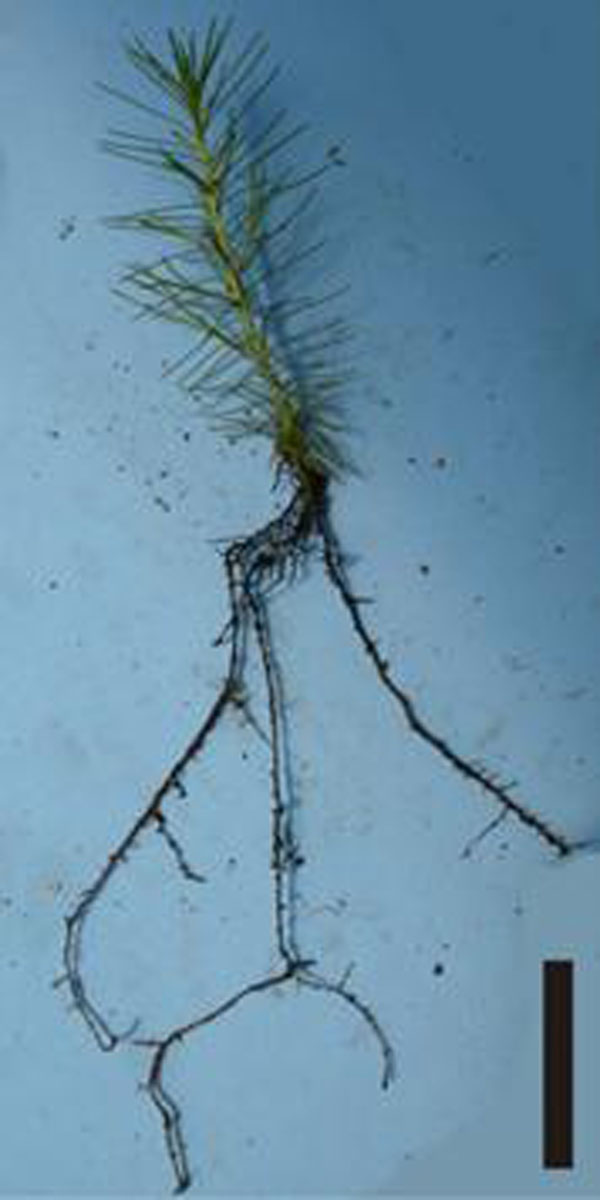
Micropropagated plants of *Pinus taeda*, 60 days after transplanted and acclimated. Bar: 5 cm.

The balance of salts in WV5 and WV3 culture media favored an optimal development of *in vitro* cultures of *Pinus taeda* due to its lower concentration of N in comparison with MS medium and to higher concentrations of thiamine and inositol, which are growth promoters. Elongated shoots were subdivided into segments, increasing the multiplication rate to 3 segments per shoot. The majority of BAP treatments did not promote better multiplication when compared to control. However, the alternate use of 0.12 μM BAP added to WV5 culture medium during initial culture and a BAP-free medium during the 1^st^ subculture can increase the multiplication rate. The estimated production was 1024 shoots from 100 explants, in seven months of cultivation. The best rooting percentage (37.5%) was obtained in shoots treated with 2.69 μM NAA and 0.44 μM BAP for 9 days in culture medium composed of water and agar without liquid phase, followed by transfer to growth regulator-free GDm/2 medium. The double-layer medium did not increase the rooting percentage. This result was higher than that found in *Pinus virginiana*, when the same combination of plant growth regulators was used [13]. The roots originated directly and indirectly from the stem with callus formation. After 90 days of acclimatization, the survival rate was 90% and an average of 4.6 roots per plant was obtained (Figure 2). This result was better than that obtained in other study with *Pinus taeda* that reported 38% of necrosis five weeks after transplantation [14]. Micropropagation of *Pinus taeda* from axillary buds and juvenile material is feasible, but requires further studies to optimize the rooting stage.
